# Evaluation of Antiplasmodial Activity of Quinoline Derivatives Incorporating Arylnitro and Aminochalcone Moieties

**DOI:** 10.3390/ph19050740

**Published:** 2026-05-08

**Authors:** Nanang R. Ariefta, Richard M. Beteck, Lesetja J. Legoabe, Yoshifumi Nishikawa

**Affiliations:** 1 National Research Center for Protozoan Diseases, Obihiro University of Agriculture and Veterinary Medicine, Inada-cho, Obihiro 080-8555, Japan; nanang.ariefta@gmail.com; 2 Department of Pharmaceutical Chemistry, Centre of Excellence for Pharmaceutical Sciences, North-West University, Private Bag X6001, Potchefstroom 2520, South Africa; richard.beteck@nwu.ac.za (R.M.B.); lesetja.legoabe@nwu.ac.za (L.J.L.)

**Keywords:** quinoline, arylnitro, aminochalcone, antiplasmodial, *Plasmodium falciparum*

## Abstract

**Background/Objectives:** The widespread emergence of chloroquine-resistant *Plasmodium falciparum* continues to drive the search for new quinoline-based antimalarial agents capable of retaining efficacy against resistant parasites. This study aimed to evaluate a series of synthetic quinoline derivatives incorporating arylnitro and aminochalcone moieties for their antiplasmodial activity and selectivity. **Methods:** A series of eighteen synthetic quinoline derivatives were evaluated for in vitro antiplasmodial activity against *P. falciparum* strains (3D7, K1, and Dd2), along with cytotoxicity in mammalian cells and hemolytic activity in human red blood cells. Structure–activity relationship analysis was performed, and molecular docking studies were conducted against β-hematin and the chloroquine resistance transporter (PfCRT). **Results:** Several compounds exhibited sub-micromolar activity against the chloroquine-sensitive 3D7 strain. The most potent compound (Compound **14**), a nitro-substituted N-alkylated quinoline bearing a CF_3_-enriched aromatic chalcone framework, demonstrated high potency and selectivity (IC_50_ = 0.13 μM; SI = 1132.92). Importantly, this compound retained substantial activity against multidrug-resistant K1 and Dd2 strains, displaying lower resistance indices than chloroquine. Structure–activity relationship analysis revealed that nitro substitution, N-alkylation, and halogen/CF_3_-rich aromatic features critically influence potency and selectivity. Docking studies suggested that Compound **14** engages both β-hematin and PfCRT more extensively than chloroquine. **Conclusions:** These findings identify Compound **14** as a promising lead scaffold for further optimization toward next-generation antimalarial agents.

## 1. Introduction

Malaria remains one of the most devastating infectious diseases worldwide, with *Plasmodium falciparum* responsible for the majority of severe cases and malaria-related deaths [1]. Despite significant advances in chemotherapy and vector control [2,3], the continued emergence and spread of drug-resistant parasites threaten current treatment strategies and underscore the urgent need for new antimalarial agents with novel or resistance-resilient mechanisms of action.

For decades, chloroquine was the cornerstone of malaria treatment owing to its low cost, excellent safety profile, and remarkable efficacy [4]. Its antimalarial activity is closely associated with inhibition of hemozoin formation in the parasite digestive vacuole, leading to toxic accumulation of free heme and parasite death [5]. However, widespread resistance to chloroquine has rendered this drug largely ineffective against *P. falciparum* in most endemic regions. This resistance is primarily attributed to mutations in the *P. falciparum* chloroquine resistance transporter (PfCRT), a transmembrane protein located in the digestive vacuole membrane that mediates the efflux of protonated chloroquine from the vacuole, thereby reducing its effective intra-parasitic concentration [6].

Despite the failure of chloroquine itself, quinoline-based scaffolds continue to play a central role in antimalarial drug discovery, as evidenced by clinically successful compounds such as amodiaquine, piperaquine, and mefloquine [7,8]. These examples illustrate that appropriate structural modification of the quinoline core can overcome resistance mechanisms and restore or even enhance antimalarial efficacy. In this context, hybrid or “dual-function” molecules that combine the quinoline pharmacophore with additional functional motifs have emerged as a promising strategy to improve potency, modulate physicochemical properties, and potentially engage multiple parasite targets [9,10,11].

Chalcone and related α,β-unsaturated carbonyl systems have attracted considerable attention in antimalarial research due to their broad biological activity profiles and synthetic versatility [12]. Numerous chalcone derivatives have been reported to display antiplasmodial activity through diverse mechanisms, including interference with heme detoxification, redox homeostasis, and parasite metabolic pathways [13]. The combination of quinoline and chalcone motifs into a single molecular framework therefore represents an attractive approach to generate multifunctional antimalarial agents with enhanced efficacy and resistance tolerance.

In the present study, we evaluate a series of eighteen synthetic quinoline derivatives incorporating arylnitro and aminochalcone moieties, which have previously exhibited efficacy against several protozoan parasites [14]. These compounds were evaluated for their in vitro antiplasmodial activity against both chloroquine-sensitive and multidrug-resistant *P. falciparum* strains, as well as for their cytotoxicity and hemolytic liability. To rationalize the observed biological activity and guide lead selection, structure–activity relationship analysis and in silico pharmacokinetic profiling were performed. In addition, molecular docking studies were conducted against both β-hematin (a synthetic hemozoin model) and PfCRT to explore potential structural bases for hemozoin growth inhibition and resistance circumvention. Through this integrated approach, we identify a lead compound that combines high potency, excellent selectivity, and sustained activity against resistant parasites, and we provide a framework for further optimization of this scaffold toward next-generation quinoline-based antimalarial agents.

## 2. Results

### 2.1. In Vitro Antiplasmodial Activity of Quinoline Derivatives Against P. falciparum 3D7

All eighteen quinoline derivatives were evaluated for their in vitro antiplasmodial activity against the chloroquine-sensitive *P. falciparum* 3D7 strain using a SYBR Green I-based growth inhibition assay. The compounds displayed a wide range of activities, with IC_50_ values spanning from 0.13 to 4.40 μM (Table 1). Several compounds exhibited sub-micromolar potency against the 3D7 strain. Among them, Compound **14** showed the highest activity, with an IC_50_ value of 0.13 ± 0.02 μM, followed by Compounds **4** (0.40 ± 0.06 μM), **18** (0.40 ± 0.22 μM), **6** (0.41 ± 0.07 μM), **13** (0.41 ± 0.03 μM), **7** (0.49 ± 0.09 μM), and **9** (0.60 ± 0.17 μM). Compounds **5** and **8** also retained sub-micromolar activity, with IC_50_ values of 0.68 ± 0.05 μM and 0.83 ± 0.02 μM, respectively. In contrast, several derivatives showed only moderate activity, including Compounds **1**–**3**, **11**, **12**, **15**–**17**, with IC_50_ values in the low micromolar range (1.39–4.40 μM). For comparison, chloroquine and artemisinin exhibited IC_50_ values of 0.026 ± 0.003 μM and 0.014 ± 0.002 μM, respectively, confirming the expected high sensitivity of the 3D7 strain to these reference antimalarials.

The cytotoxicity of the compounds was assessed using human skin fibroblast (HSF) cells, allowing calculation of selectivity indices (SI = CC_50_/IC_50_). Several compounds showed favorable selectivity profiles. Notably, Compound **14** combined the highest antiplasmodial potency with very low cytotoxicity (CC_50_ = 147.28 ± 91.31 μM), resulting in an exceptionally high SI (1132.92). High selectivity was also observed for Compounds **4** (SI > 500), **7** (SI = 245.22), **8** (SI = 174.73), **15** (SI = 92.62), **9** (SI = 79.42), **17** (SI = 75.67), **11** (SI = 64.47), and **16** (SI = 53.91). In contrast, Compounds **5**, **10**, **12**, and **13** exhibited relatively low SI (<25), reflecting higher cytotoxicity toward mammalian cells.

Additionally, hemolytic activity against human red blood cells (RBCs) was negligible for all tested compounds at 100 μM, with hemolysis rates below 0.1% in all cases. These values were substantially lower than those observed for chloroquine (0.71 ± 0.35%) and artemisinin (1.03 ± 0.46%), indicating that none of the tested derivatives caused appreciable erythrocyte membrane damage under the assay conditions. Importantly, the hemolysis assessment was conducted at a concentration significantly higher than the observed antiplasmodial IC_50_ values, representing a stringent condition to evaluate potential cytotoxic effects on host erythrocytes. The minimal hemolytic activity observed under these conditions suggests a favorable safety margin and supports the selectivity of the compounds toward *P. falciparum*. Higher concentrations were not evaluated due to the risk of solvent-induced membrane disruption, which could confound interpretation of compound-specific effects. Therefore, the selected concentration provides a conservative and reliable estimate of hemolytic liability.

### 2.2. Activity Profiles Against Multi-Drug-Resistant Strains of P. falciparum

Based on their potent activity and favorable selectivity against the 3D7 strain, four representative compounds (**4**, **7**, **8**, and **14**) were further evaluated against two multidrug-resistant *P. falciparum* strains, K1 and Dd2. Their antiplasmodial activities, cytotoxicities, resistance indices (RI), and SI are summarized in Table 2. All four compounds retained measurable activity against both resistant strains, although reduced potency relative to 3D7 was observed. Compound **14** remained the most active derivative, with IC_50_ values of 0.62 ± 0.18 μM against K1 and 0.33 ± 0.05 μM against Dd2, corresponding to resistance indices of 4.77 and 2.54, respectively. Compounds **4**, **7**, and **8** also showed low-micromolar activity against both resistant strains, with IC_50_ values ranging from 1.08 to 2.33 μM. Compared with chloroquine, which exhibited a marked loss of activity against K1 and Dd2 (IC_50_ = 0.740 ± 0.095 μM and 0.895 ± 0.039 μM, respectively; RI = 28.46 and 34.42), all four test compounds showed substantially lower resistance indices. In particular, Compound **14** displayed a much smaller shift in activity between sensitive and resistant strains than chloroquine.

The four compounds exhibited favorable cytotoxicity profiles, as presented in Table 1. Accordingly, high selectivity indices were maintained across all strains. Notably, Compound **14** showed exceptionally high selectivity, with SI values of 237.55 against K1 and 446.30 against Dd2. Compounds **7** and **8** also retained SI values above 60 against both resistant strains, while Compound **4** exhibited SI values greater than 85 against K1 and Dd2. For comparison, artemisinin retained high potency against all strains, with only minimal changes in IC_50_ values, whereas chloroquine showed a pronounced loss of activity against the resistant parasites.

### 2.3. Structure–Activity Relationship Study

**Figure 1:**
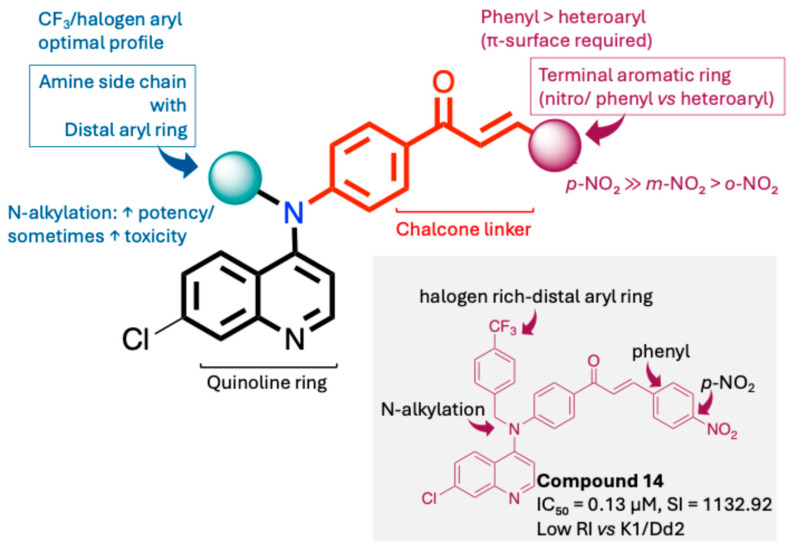
Summary of the structure–activity relationships (SAR) of the quinoline derivatives incorporating arylnitro and aminochalcone moieties and identification of Compound **14** as the optimized lead structure. The central scaffold highlights the principal variable regions, including the amine side chain with distal aryl substitution, the chalcone linker, and the terminal aromatic ring. Key SAR trends include the dominant positional effect of the nitro substituent on the terminal aromatic ring (*p*-NO_2_ ≫ *m*-NO_2_ > *o*-NO_2_), the superiority of phenyl over heteroaryl rings reflecting the requirement for an extended π-surface, the general increase in potency upon *N*-alkylation of the amine moiety (sometimes accompanied by increased cytotoxicity), and the marked benefit of CF_3_/halogen-rich distal aryl substitution. The ↑ mark indicates increased activity. The optimal combination of these features is exemplified by Compound **14**, which displays sub-micromolar antiplasmodial potency, high selectivity index (SI), and low resistance indices (RI) against the K1 and Dd2 strains.

The structure–activity relationships of the quinoline–chalcone hybrids were analyzed based on variations in the substitution pattern on the terminal aromatic ring and the amine moiety, using the in vitro antiplasmodial activity against *P. falciparum* 3D7, cytotoxicity toward HSF cells, and selectivity index as primary parameters (Figure 1).

A clear positional effect of the nitro substituent on the terminal phenyl ring was observed. In the secondary amine subseries, Compounds **3** and **4**, which are regio-isomers differing only in the position of the nitro group, showed a marked difference in activity. While Compound **3** displayed moderate activity (*ortho*-NO_2_, IC_50_ = 2.52 μM), relocation of the nitro group in Compound **4** resulted in a pronounced improvement in potency (*para*-NO_2_, IC_50_ = 0.40 μM), accompanied by excellent selectivity (SI > 500). A similar but less pronounced trend was observed in the *N*-alkylated subseries, where the *meta*-NO_2_ analogue (Compound **7**; IC_50_ = 0.49 μM) was more potent and selective than the *ortho*-NO_2_ analogue (Compound **8**; IC_50_ = 0.83 μM). These results indicate that the spatial orientation of the nitro group plays a critical role in determining the optimal binding geometry of the scaffold.

In addition to substitution pattern and amine modification, replacement of the terminal phenyl ring with five-membered heteroaromatic rings (e.g., oxygen- or sulfur-containing heterocycles) also influenced antiplasmodial activity. In general, compounds bearing heteroaryl terminal rings (e.g., Compounds **11**, **12**, **15**–**17**, and **18**) showed only low-micromolar to moderate activity and/or reduced selectivity compared with their phenyl-based counterparts. In contrast, derivatives retaining a phenyl or heavily substituted phenyl ring more frequently achieved sub-micromolar potency and higher selectivity indices. This trend suggests that an extended, hydrophobic aromatic surface is beneficial for activity, likely by promoting more favorable π–π stacking and surface complementarity with the biological target(s). The reduced activity of the heteroaryl analogues may therefore reflect a diminished ability to engage in extended aromatic stacking interactions or altered electronic and geometric properties that are less compatible with the preferred binding environment.

Comparison between the secondary aniline derivatives (Compounds **1**–**4**) and the *N*-alkylated analogues (Compounds **5**–**17**) revealed that introduction of a tertiary amine generally enhances antiplasmodial potency. Whereas most secondary amine derivatives showed low micromolar activity, several *N*-alkylated compounds exhibited sub-micromolar IC_50_ values (e.g., Compounds **5**–**10**, **13**, and **14**). This trend suggests that increased hydrophobic surface area and conformational adaptability associated with *N*-alkylation favor productive interactions with the biological target(s). However, this modification also tended to increase cytotoxicity in some cases (e.g., Compounds **5**, **6**, **10**, and **13**), indicating that potency gains must be balanced against host-cell tolerance.

Lastly, within the *N*-alkylated series, compounds bearing more extensively substituted aromatic systems, particularly those incorporating halogenated or trifluoromethyl groups, showed a further enhancement in potency. This trend culminated in Compound **14**, which combines a nitro-substituted aromatic ring, a tertiary amine, and a halogen/CF_3_-rich aryl moiety. This compound exhibited the highest potency in the series (IC_50_ = 0.13 μM), low cytotoxicity (CC_50_ = 147.28 μM), and the highest selectivity index (SI = 1132.92). Importantly, Compound **14** also retained activity against chloroquine-resistant strains with low resistance indices, indicating that this substitution pattern confers not only high potency but also robustness against resistance mechanisms.

### 2.4. In Silico Pharmacokinetic and Drug-Likeness Prediction

The in silico pharmacokinetic and drug-likeness properties of the most potent compound, Compound **14**, were evaluated using SwissADME and Deep-PK, and the results are summarized in Table 3. Compound **14** exhibited a molecular weight of 587.98 g/mol, exceeding the conventional 500 g/mol threshold. However, several other key physicochemical parameters were within commonly accepted ranges for oral drug candidates, including the number of hydrogen bond acceptors (7), number of rotatable bonds (9), topological polar surface area (TPSA = 79.02 Å^2^), and lipophilicity (LogP = 4.16). In terms of absorption-related properties, Compound **14** was predicted to be orally bioavailable and to possess adequate intestinal absorption. Notably, Compound **14** was predicted to be non-permeable to the blood–brain barrier (BBB), suggesting limited central nervous system exposure. This property may reduce the risk of central nervous system-related adverse effects but could limit direct applicability in treating cerebral malaria. The predicted total human clearance was 5.84 mL/min/kg. The estimated acute oral toxicity in rats (LD_50_) was 2.77 (log[1/(mol/kg)]). However, the compound was predicted to be positive in the AMES mutagenicity test and to have a relatively low maximum tolerated dose in humans (−0.3 log mL/min/kg), suggesting potential safety liabilities. Consistent with these physicochemical features, drug-likeness evaluation using multiple rule-based filters showed that Compound **14** does not fully comply with the Lipinski, Ghose, Egan, or Muegge criteria, which collectively define preferred physicochemical space for orally active small molecules. In contrast, it satisfies the Veber rule, reflecting acceptable polarity and molecular flexibility. Overall, these findings indicate that while Compound **14** is not “drug-like” compound, its absorption-related descriptors remain compatible with oral exposure, supporting its consideration as a lead scaffold requiring further structural optimization.

### 2.5. Molecular Docking Against β-Hematin (Hemozoin)

**Figure 2:**
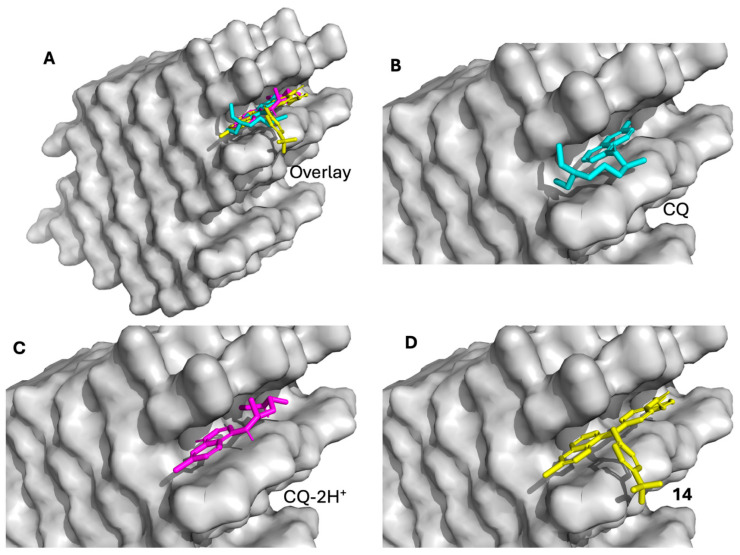
Molecular docking of chloroquine (CQ), diprotonated chloroquine (CQ-2H^+^), and Compound **14** on the β-hematin (hemozoin) crystal surface. (**A**) Overlay of CQ (cyan), CQ-2H^+^ (magenta), and Compound **14** (yellow) showing that all three ligands bind within the same surface groove of the β-hematin lattice. (**B**) Surface adsorption mode of CQ. (**C**) Surface adsorption mode of CQ-2H^+^. (**D**) Binding mode of Compound **14**, which adopts an extended surface-adsorbed conformation spanning a substantially larger area of the porphyrin lattice than the reference compounds.

Molecular docking simulations were performed to compare the binding behavior of chloroquine (CQ), diprotonated chloroquine (CQ-2H^+^, included to reflect the physiological protonation state of CQ within the acidic digestive vacuole of *P. falciparum*, where the drug predominantly exists in diprotonated forms), and Compound **14** on the β-hematin crystal model. All three ligands were predicted to bind within the same surface pocket located on the exposed porphyrin lattice of the β-hematin supercell (Figure 2), consistent with a surface adsorption ligand interaction. The docking scores are summarized in Table 4. Compound **14** exhibited a substantially stronger predicted binding affinity (−13.55 kcal/mol) than both CQ (−7.50 kcal/mol) and CQ-2H^+^ (−8.73 kcal/mol), while maintaining a high CNN score (0.8281), indicating a reliable binding pose. This CNN score, derived from convolutional neural network-based rescoring of docking poses, reflects the likelihood that the predicted ligand orientation represents a physically realistic binding mode. In contrast, CQ and CQ-2H^+^ showed weaker predicted affinities, despite slightly higher CNN scores. Visual inspection of the docking poses revealed that all three ligands associate with the same surface groove of the β-hematin lattice (Figure 2A); however, their binding modes differ markedly in surface coverage and extent of interaction with the porphyrin plane. CQ and CQ-2H^+^ adopt relatively compact, shallow adsorption modes, interacting with a limited area of the crystal surface (Figure 2B,C). In comparison, Compound **14** spans a broader region of the exposed porphyrin surface and aligns more extensively along the crystal groove (Figure 2D), enabling increased aromatic surface complementarity and enhanced π–π stacking interactions. This extended surface adsorption mode of Compound **14** may contribute to its substantially higher predicted binding affinity and, when considered alongside the in vitro activity data, its potent antiplasmodial effect. However, these docking results represent static binding models and do not directly establish inhibition of β-hematin formation. Rather, they provide a structural basis for understanding how Compound **14** may interact more effectively with the hemozoin surface compared to chloroquine.

### 2.6. Molecular Docking Against PfCRT

**Figure 3:**
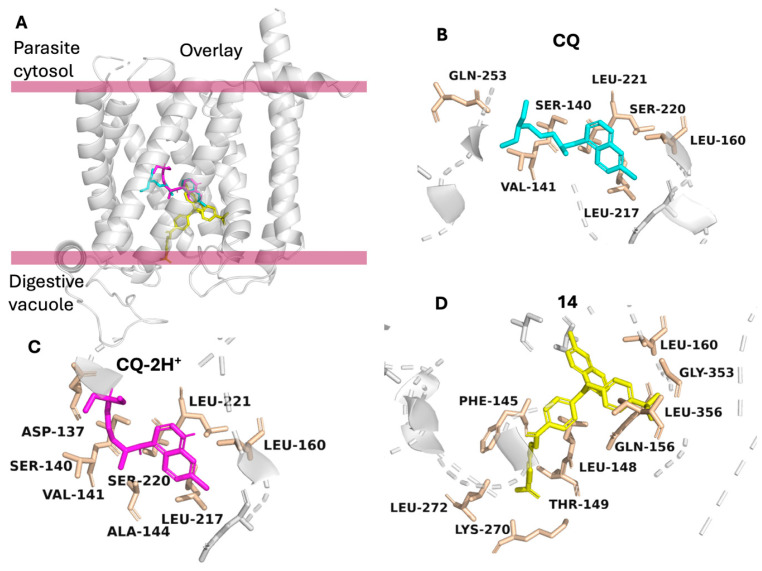
Molecular docking of chloroquine (CQ), diprotonated chloroquine (CQ-2H^+^), and Compound **14** in the central cavity of the *Plasmodium falciparum* chloroquine resistance transporter (PfCRT; PDB ID: 6UKJ). (**A**) Overall view of PfCRT in a membrane-spanning orientation, showing the location of the central cavity and the binding region of the three ligands (CQ, cyan; CQ-2H^+^, magenta; Compound **14**, yellow). The approximate membrane boundaries are indicated by horizontal magenta bars. (**B**) Binding mode of CQ. (**C**) Binding mode of CQ-2H^+^. (**D**) Binding mode of Compound **14**, which adopts a more extended conformation and engages a broader network of interactions.

To further explore whether the potent antiplasmodial activity of Compound **14** could be associated with direct interaction with the chloroquine resistance transporter, molecular docking simulations were performed against PfCRT (PDB ID: 6UKJ). Docking results for CQ, CQ-2H^+^, and Compound **14** are summarized in Table 4. Compound **14** exhibited a markedly stronger predicted binding affinity toward PfCRT (−9.07 kcal/mol) than both CQ (−5.93 kcal/mol) and CQ-2H^+^ (−5.41 kcal/mol), while maintaining a high CNN score (0.8681), indicating a reliable binding pose. In contrast, the reference compounds showed weaker predicted affinities despite similarly high CNN scores. Analysis of the docking poses revealed that all three ligands bind within the central cavity of PfCRT (Figure 3A). However, their interaction patterns differed substantially (Figure 3B–D). CQ formed hydrogen bond interactions with Ser140, Ser220, and Gln253, and was stabilized primarily by hydrophobic contacts with Val141, Leu160, Leu217, and Leu221. CQ-2H^+^ exhibited hydrogen bonding with Asp137, Ser140, and Ser220, and additional hydrophobic interactions involving Val141, Ala144, Leu160, Leu217, and Leu221. In contrast, Compound **14** adopted a distinct and more extensive binding mode within the PfCRT cavity. It formed a hydrogen bond with Lys270 and established multiple hydrophobic interactions with residues lining the cavity, including Phe145, Leu148, Thr149, Gln156, Leu160, Leu272, and Leu356. In addition, a halogen bond interaction with Gly353 was observed, further stabilizing the complex. This broader interaction network and deeper engagement within the cavity may contribute to the substantially improved binding affinity of Compound **14** compared with the reference compounds. Notably, several of the interacting residues are located in or near regions known to harbor resistance-associated mutations, including the canonical K76T substitution and other mutations such as C72S, A220S, N326D, and I356L [15], supporting the relevance of this binding site for modulation of PfCRT-mediated drug interactions. However, these docking results describe static binding compatibility and do not by themselves establish the functional consequence of binding; specifically, they cannot distinguish whether Compound **14** behaves as a transported substrate, an inhibitor, or interacts in a non-transported manner.

## 3. Discussion

The emergence and global spread of chloroquine-resistant *P. falciparum* remain a major obstacle to malaria control and continues to motivate the search for new quinoline-based scaffolds that can retain efficacy against resistant parasites while maintaining acceptable safety profiles [8]. In this study, we report a series of quinoline derivatives incorporating arylnitro and aminochalcone moieties that combine potent antiplasmodial activity with low hemolytic liability and, in selected cases, high selectivity toward the parasite over mammalian cells.

Among the eighteen synthesized derivatives, several compounds exhibited sub-micromolar activity against the chloroquine-sensitive 3D7 strain, with Compound **14** emerging as a clear lead, combining the highest potency (IC_50_ = 0.13 μM) with exceptional selectivity (SI > 1000) and negligible hemolytic activity. Importantly, Compound **14** retained substantial activity against the multidrug-resistant K1 and Dd2 strains, displaying markedly lower resistance indices than chloroquine. This behavior is particularly noteworthy, as classical 4-aminoquinolines typically show dramatic losses of activity against PfCRT-mutant parasites due to active drug efflux from the digestive vacuole [5,6].

The SAR analysis revealed four dominant structural determinants of activity: (i) the positional orientation of the nitro substituent on the terminal aromatic ring, (ii) the nature of the terminal aromatic system (phenyl vs. heteroaryl), (iii) the presence of a tertiary amine, and (iv) the incorporation of a halogen/CF_3_-rich distal aromatic moiety. Hybrid quinoline–chalcone scaffolds have been pursued as antimalarial agents because combining pharmacophores often yields improved potency and modulated biological profiles, as demonstrated in multiple quinoline–chalcone and quinoline hybrid series showing enhanced activity relative to parent scaffolds [9]. The strong regio-isomer effects observed for the nitro group (e.g., Compounds **3** vs. **4** and **7** vs. **8**) indicate that subtle geometric changes strongly influence productive target engagement, consistent with a binding mode that depends on surface complementarity rather than simple electrostatic interactions. Substitution effects in related chalcone derivatives have also been shown to affect activity through changes in electronics and geometry [16]. In parallel, replacement of the phenyl ring with five-membered heteroaromatic systems generally led to reduced potency and/or selectivity, highlighting the importance of an extended hydrophobic π-surface for optimal activity; such aromatic surface complementarity has been noted as a key factor in chalcone and quinoline hybrid SAR [17]. The general improvement in potency upon *N*-alkylation suggests that increased hydrophobic surface area and conformational adaptability enhance biological activity, although the accompanying increase in cytotoxicity in some analogues highlights the narrow optimization window typical of amphiphilic antimalarial scaffolds.

The in silico docking results provide a plausible structural framework for interpreting these trends. At the level of hemozoin, Compound **14** shows a much stronger predicted binding affinity than chloroquine or its diprotonated form and adopts an extended surface-adsorbed pose spanning a larger area of the porphyrin lattice. This mode of interaction is consistent with established models in which quinoline antimalarials inhibit β-hematin growth by adsorbing to specific crystal faces or growth steps, thereby blocking further crystal elongation [18]. The enhanced aromatic surface coverage and π–π stacking potential of Compound **14** may contribute to its superior predicted affinity and, when considered alongside the in vitro data, its high in vitro potency, a relationship that has also been noted in previous hemozoin-binding and docking studies of quinoline derivatives [19]. In parallel, docking to PfCRT suggests that Compound **14** can engage the central cavity of the transporter more extensively than chloroquine, forming a broader network of hydrophobic, hydrogen-bonding, and halogen-bond interactions, including contacts near residues associated with resistance mutations [15]. As docking describes static binding compatibility, it cannot by itself establish whether such interactions correspond to transport, inhibition, or both. However, these structural observations can be interpreted alongside the in vitro resistance indices. Chloroquine displayed a pronounced resistance shift (RI = 28.46 in K1 and 34.42 in Dd2), consistent with efficient PfCRT-mediated efflux. In contrast, Compound **14** showed only a modest shift (RI = 4.77 in K1 and 2.54 in Dd2), indicating substantially reduced susceptibility to PfCRT-associated resistance mechanisms. Taken together, these findings suggest that Compound **14** interacts with PfCRT in a manner that may reduce its susceptibility to efflux relative to chloroquine. Importantly, this interpretation does not imply that Compound **14** functions as a PfCRT inhibitor; rather, it supports the possibility that its binding mode or physicochemical properties limit its recognition or transport efficiency. However, this remains a hypothesis, and direct experimental studies will be required to determine whether Compound **14** acts as a transported substrate, an inhibitor, or both. Despite these promising features, several limitations must be acknowledged. First, the mechanistic interpretation remains inferential and is based on docking models rather than direct biochemical or biophysical measurements. Second, the present study is limited to in vitro parasite assays, and the translation of these findings to in vivo efficacy and safety remains to be established. Third, the in silico ADME analysis indicates that Compound **14** exceeds classical molecular weight guidelines and shows potential liabilities related to mutagenicity and tolerability, highlighting the need for further optimization of its drug-like properties.

Future work should therefore focus on several directions. As an immediate priority, in vivo evaluation of Compound **14** in suitable malaria models will be essential to assess its pharmacokinetic behavior, tolerability, and therapeutic efficacy. Based on these outcomes, subsequent SAR-guided optimization may focus on improving drug-likeness properties, including reducing molecular weight and enhancing pharmacokinetic profiles, while preserving the favorable binding characteristics of Compound **14**. From a mechanistic standpoint, direct assays of β-hematin inhibition and functional studies of PfCRT-mediated transport will be important to test the hypotheses suggested by the docking models.

## 4. Materials and Methods

### 4.1. Chemicals

Eighteen quinoline-based derivatives incorporating arylnitro and aminochalcone moieties (Table 1) were synthesized according to previously published methods [14]. Chloroquine diphosphate (CQ) and artemisinin (ART) were obtained from Sigma-Aldrich (St. Louis, MO, USA) and used as reference compounds. Dimethyl sulfoxide (DMSO; Wako, Osaka, Japan) was used as the vehicle control.

### 4.2. P. falciparum In Vitro Culture

In vitro assays were performed using three *P. falciparum* strains: 3D7 (chloroquine-sensitive) and K1 and Dd2 (both multidrug-resistant). Parasites were maintained in RPMI-1640 medium (Sigma-Aldrich) supplemented with 2% *v*/*v* O(+) erythrocytes (supplied by the Japanese Red Cross Society, Sapporo, Japan), 0.5% *w*/*v* AlbuMax™ II (Gibco, Waltham, MA, USA), 25 mM HEPES (Sigma-Aldrich), 24 mM NaHCO_3_ (Wako), 184 μM hypoxanthine (Wako), and 0.025% *v*/*v* gentamicin (Gibco), and cultured at 37 °C under an atmosphere of 90% N_2_, 5% CO_2_, and 5% O_2_. Parasitemia was routinely determined by microscopic examination of Giemsa-stained thin blood smears.

### 4.3. Growth Inhibition Assay of P. falciparum

Highly synchronized ring-stage parasites (>90%, obtained by the sorbitol method) were dispensed into 96-well plates and exposed to eight twofold serial dilutions of the test compounds (20 mM stock solutions), giving final concentrations ranging from 10 to 0.078 μM. Each well received 50 μL of the compound solution and 50 μL of parasite suspension (0.5% parasitemia, 2% hematocrit). Following incubation for 72 h, 100 μL of SYBR Green I solution (0.02% *v*/*v* in lysis buffer; Lonza, Basel, Switzerland) was added to each well. The plates were then incubated in the dark at room temperature for 2 h without freezing, and fluorescence was measured at excitation/emission wavelengths of 485/518 nm using a SpectraMax iD5 microplate reader (Molecular Devices, San Jose, CA, USA). Data are presented as the means of at least three independent experiments, each performed with four technical replicates. IC_50_ values were determined by nonlinear regression analysis using GraphPad Prism 9.

### 4.4. Cytotoxicity and Hemolysis Assays

Human skin fibroblasts cells (HSF; NB1RGB, RCB0222, RIKEN BRC, Tsukuba, Japan) were maintained in DMEM (Sigma) supplemented with 10% fetal bovine serum (FBS; Serana, Brandenburg, Germany) and 1% penicillin–streptomycin (Wako). Cells were seeded into 96-well plates at a density of 1 × 10^4^ cells per well in 100 μL of medium and allowed to adhere for 48 h. The cultures were then treated with 100 μL of serially diluted test compound solutions (20 mM stock solutions), yielding final concentrations ranging from 200 to 1.56 μM, and incubated for an additional 72 h. Cell viability was determined by adding 5 μL of Cell Counting Kit-8 (CCK-8; Dojindo, Kumamoto, Japan) to each well, followed by incubation for 3 h at 37 °C. Absorbance was measured at 450 nm. CC_50_ values were determined by nonlinear regression analysis using GraphPad Prism 9. Hemolytic activity against human red blood cells was evaluated as described previously [20].

### 4.5. Pharmacokinetics Prediction and Molecular Docking

SMILES strings for the compounds were generated using ChemDraw 16. Pharmacokinetic properties were evaluated using SwissADME [21] and Deep-PK [22]. For molecular docking simulations, conformers of Compound **14** ((*E*)-1-(4-((7-chloroquinolin-4-yl)(4-(trifluoromethyl)benzyl)amino)phenyl)-3-(4-nitrophenyl)prop-2-en-1-one) were generated using RDKit with the ETKDGv3 algorithm (Experimental Torsion Knowledge Distance Geometry, version 3) [23] and subsequently optimized using the MMFF (Merck Molecular Force Field). The resulting conformations were clustered by DBSCAN (Density-Based Spatial Clustering of Applications with Noise) [24,25] to group similar geometries and select representative ligand poses, yielding ten clusters.

Molecular docking was performed for each representative conformer against both β-hematin and PfCRT using Gnina [26,27,28,29]. The crystal structure of β-hematin was obtained from the Cambridge Crystallographic Data Centre (CCDC; deposition number 162267) [30]. The β-hematin structure was expanded into a hemozoin supercell (2 × 2 × 2 lattice) using VESTA software Version 3 [31] and converted to PDB format for docking. The docking grid was defined to encompass the entire hemozoin receptor. The ligand–receptor complex showing the most favorable convolutional neural network (CNN) score and binding energy was selected for further analysis, and the interactions were visualized in three dimensions using PyMOL Version 3.1.6.1.

The structure of the *P. falciparum* chloroquine resistance transporter (PfCRT) was retrieved from the RCSB Protein Data Bank (PDB ID: 6UKJ), corresponding to the cryo-EM structure of the chloroquine-resistant 7G8 isoform. This PfCRT is a 10-transmembrane helix transporter that contains a large, negatively charged central cavity, which represents the principal binding and transport site for protonated 4-aminoquinoline antimalarials and is lined by multiple resistance-associated residues, including the essential K76T mutation and additional substitutions such as C72S, A220S, N326D, and I356L [15]. Docking grids were defined to cover the entire receptor. Protein–ligand interactions were analyzed using PLIP (Protein–Ligand Interaction Profiler; https://plip-tool.biotec.tu-dresden.de/plip-web/plip/index, accessed on 16 January 2026) [32]. Chloroquine (CQ) and diprotonated chloroquine (CQ-2H^+^) were included as reference compounds. PyMOL Version 3.1.6.1 was used for molecular visualization and figure preparation.

## 5. Conclusions

In summary, this study identifies Compound **14** as a quinoline-based lead scaffold with potent antiplasmodial activity against both chloroquine-sensitive and multidrug-resistant *P. falciparum* strains, along with a favorable selectivity profile. While the mechanistic and pharmacokinetic insights presented here are based on computational analyses and remain to be experimentally validated, the observed resistance profile and in vitro efficacy highlight the potential of this scaffold for further development. These findings provide a foundation for future in vivo evaluation, mechanistic studies, and rational optimization toward next-generation antimalarial agents.

## Figures and Tables

**Table 1:** Summary of the in vitro activities of 18 quinoline derivatives incorporating arylnitro and aminochalcone moieties tested in this study against *P. falciparum* 3D7, HSF cells, and human RBCs.

Comp.	Structure	IC_50_ *P. falciparum* 3D7 (μM)	CC_50_ HSF Cells (μM)	SI	Human RBCs Hemolysis Rate at 100 μM (%)
**1**	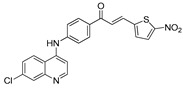	2.32 ± 0.97	>200.00	>86.21	0.010 ± 0.005
**2**	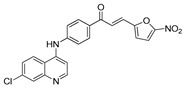	2.59 ± 1.06	83.32 ± 40.93	32.17	0.009 ± 0.004
**3**	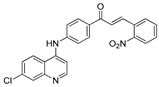	2.52 ± 0.68	>200.00	>79.37	0.018 ± 0.003
**4**	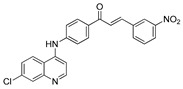	0.40 ± 0.06	>200.00	>500.00	0.006 ± 0.003
**5**	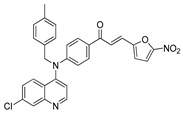	0.68 ± 0.05	9.92 ± 3.35	14.59	0.006 ± 0.003
**6**	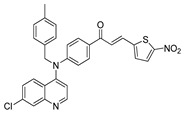	0.41 ± 0.07	13.15 ± 3.83	32.07	0.025 ± 0.005
**7**	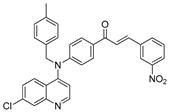	0.49 ± 0.09	120.16 ± 35.78	245.22	0.045 ± 0.011
**8**	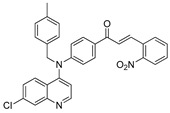	0.83 ± 0.02	145.03 ± 67.42	174.73	0.019 ± 0.011
**9**	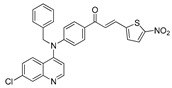	0.60 ± 0.17	47.65 ± 16.97	79.42	0.056 ± 0.011
**10**	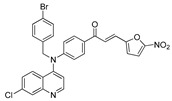	1.03 ± 0.09	14.03 ± 4.18	13.62	0.005 ± 0.003
**11**	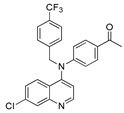	1.41 ± 0.18	90.90 ± 33.27	64.47	0.067 ± 0.011
**12**	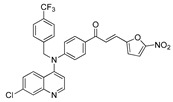	4.40 ± 0.88	36.34 ± 9.68	8.26	0.003 ± 0.002
**13**	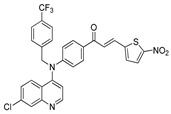	0.41 ± 0.03	10.10 ± 3.74	24.63	0.041 ± 0.014
**14**	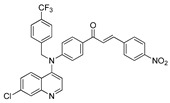	0.13 ± 0.02	147.28 ± 91.31	1132.92	0.043 ± 0.009
**15**	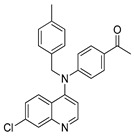	1.63 ± 0.33	150.97 ± 37.12	92.62	0.061 ± 0.013
**16**	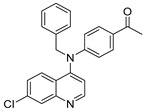	2.91 ±0.29	156.88 ± 57.84	53.91	0.032 ± 0.007
**17**	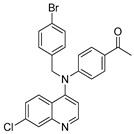	1.39 ± 0.09	105.18 ± 23.64	75.67	0.052 ± 0.011
**18**	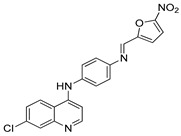	0.40 ± 0.22	18.32 ± 3.71	45.80	0.002 ± 0.001
Chloroquine (CQ)		0.026 ± 0.003	20.71 ± 6.8	796.54	0.71 ± 0.35
Artemisinin (ART)		0.014 ± 0.002	153.00 ± 30.76	10,928.57	1.03 ± 0.46

Values are presented as the average ± SD of at least three independent experiments. IC_50_, half-maximal inhibitory concentration. CC_50_, half-maximal cytotoxic concentration. SI, selectivity index (ratio between IC_50_ and CC_50_). HSF, human skin fibroblast cell; RBC, red blood cell.

**Table 2:** Summary of the in vitro activities of top four quinoline derivatives incorporating arylnitro and aminochalcone moieties tested in this study against *P. falciparum* 3D7, K1, Dd2, and HSF cells.

Comp.	IC_50_ *P. falciparum* (μM)			CC_50_ HSF Cells (μM)	RI		SI		
	3D7	K1	Dd2		K1	Dd2	3D7	K1	Dd2
**4**	0.40 ± 0.06	2.33 ± 0.39	1.52 ± 0.16	>200.00	5.83	3.80	>500.00	>85.84	>131.58
**7**	0.49 ± 0.09	1.79 ± 0.88	1.08 ± 0.14	120.16 ± 35.78	3.65	2.20	245.22	67.13	111.26
**8**	0.83 ± 0.02	1.66 ± 0.54	1.31 ± 0.20	145.03 ± 67.42	2.00	1.58	174.73	87.37	110.71
**14**	0.13 ± 0.02	0.62 ± 0.18	0.33 ± 0.05	147.28 ± 91.31	4.77	2.54	1132.92	237.55	446.30
CQ	0.026 ± 0.003	0.740 ± 0.095	0.895 ± 0.039	20.71 ± 6.8	28.46	34.42	796.54	27.99	23.14
ART	0.014 ± 0.002	0.019 ± 0.002	0.046 ± 0.008	153.00 ± 30.76	1.36	3.29	10,928.57	8052.63	3326.09

Values are presented as the average ± SD of at least three independent experiments. RI, resistance index, the ratio between the IC_50_ values of *P. falciparum* K1or Dd2 and 3D7. SI, selectivity index (ratio between IC_50_ and CC_50_). HSF, human skin fibroblast cell.

**Table 3:** Predicted pharmacokinetic properties of Compounds **14**.

Parameter	Compound 14	Desired Value
Molecular Weight (g/mol)	587.98	≤500
No. H-bond acceptors	7	≤10
No. H-bond donors	0	≤5
No. rotatable bond (rotors)	9	≤10
Topological polar surface area (TPSA, Å^2^)	79.02	≤140
LogP octanol/water partition coefficient	4.16	≤5
Human Oral Bioavailability 50%	Bioavailable	
BBB permeant	No	
Intestinal absorption	Absorbed	
Total clearance (mL/min/kg)	5.84	
LD50 oral rat acute toxicity (log[1/(mol/kg)])	2.77	
AMES mutagenesis	Toxic	
Max tolerated dose in human (log mL/min/kg)	−0.3	≥0.477 high tolerance
Drug likeness		
Lipinski (Pfizer)	No	
Ghose	No	
Veber (GSK)	Yes	
Egan (Pharmacia)	No	
Muegge (Bayer)	No	

**Table 4:** Interactions of CQ, CQ-2H^+^, and Compound **14** from docking simulations.

Receptor	Ligand		
	CQ	CQ-2H^+^	Compound 14
β-hematin			
Binding affinity (kcal/mol)	−7.50	−8.73	−13.55
CNN score	0.9451	0.9319	0.8281
PfCRT (6UKJ)			
Binding affinity (kcal/mol)	−5.93	−5.41	−9.07
CNN score	0.8053	0.8705	0.8681
Hydrogen bond interactions	Ser140, Ser220, Gln253	Asp137, Ser140, Ser220	Lys270
Hydrophobic interactions	Val141, Leu160, Leu217, Leu221	Asp137, Val141, Ala144, Leu160, Leu217, Leu221	Phe145, Leu148, Thr149, Gln156, Leu160, Leu272, Leu356
Halogen Bond			Gly353

## Data Availability

The original contributions presented in this study are included in the article. Further inquiries can be directed to the corresponding author.

## Abbreviations

The following abbreviations are used in this manuscript:

ADME	Absorption, Distribution, Metabolism, and Excretion
ART	Artemisinin
BBB	Blood-brain barrier
CC_50_	50% cytotoxic concentration
CF_3_	Trifluoromethyl
CNN	Convolutional Neural Network
CQ	Chloroquine
CQ-2H^+^	Diprotonated chloroquine
DBSCAN	Density-Based Spatial Clustering of Applications with Noise
DMSO	Dimethyl sulfoxide
ETKDG	Experimental Torsion Knowledge Distance Geometry
FBS	Fetal bovine serum
HSF	Human skin fibroblasts cell
IC_50_	50% inhibitory concentration
LD_50_	Median lethal dose
MMFF	Merck Molecular Force Field
MW	Molecular weight
PfCRT	*Plasmodium falciparum* chloroquine resistance transporter
PDB	Protein Data Bank
PLIP	Protein–Ligand Interaction Profiler
RI	Resistance index
SAR	Structure–activity relationship
SI	Selectivity index
TPSA	Topological polar surface area
